# Boosting Creativity, but Only for Low Creative Connectivity: The Moderating Effect of Priming Stereotypically Inconsistent Information on Creativity

**DOI:** 10.3389/fpsyg.2019.00273

**Published:** 2019-02-11

**Authors:** Fangfang Wen, Bin Zuo, Zhijie Xie, Jia Gao

**Affiliations:** ^1^School of Psychology, Central China Normal University, Wuhan, China; ^2^Center for Studies of Social Psychology, Central China Normal University, Wuhan, China; ^3^Key Laboratory of Adolescent Cyberpsychology and Behavior, Ministry of Education, Wuhan, China

**Keywords:** individual creativity, stereotypically inconsistent information, creativity connectivity, poster design task, priming paradigm

## Abstract

Previous researchers have documented that priming inconsistent stereotypic information boosts creativity. The current study further examined the moderating role of creativity connectivity—which is the degree to which people perceive a social group or professional role to be relevant to creativity—in the priming of information related to the boosting effects of creativity. Study 1 adopted a 2 (stereotypically inconsistent target gender: male vs. female) × 2 [priming types: stereotypically consistent information (SCI) priming vs. stereotypically inconsistent information (SICI) priming] group design in which 89 college students from Wuhan were enrolled to complete a priming paradigm and a poster-advertising-design task. As a result, we found that the activation of inconsistent stereotypic information boosted creativity compared with that of consistent stereotypic information, which replicated previous findings. Study 2 also adopted a 2 (creativity-domain connectivity: high vs. low) × 2 (priming types: SCI priming vs. SICI priming) group design in which 85 college students from Wuhan were enrolled to complete the same tasks as in Study 1. The results of Study 2 indicated that when information with low relevance to creativity such as “a nurse” was primed, creativity was then significantly boosted by inconsistent stereotypic information such as “a male nurse” compared with the stereotypic one such as “a female nurse.” Conversely, when information with high relevance to creativity such as “a poet” was primed, there were no significant creativity-boosting effects between inconsistent stereotypic information such as “a dull poet” and the consistent one such as “an eccentric poet.” In sum, this study (i) replicated the previous findings in Chinese culture and (ii) further explored the moderating role of creativity connectivity of the inconsistent stereotypic information.

## Introduction

Creativity, which refers to the ability of individuals to generate novel and useful products (Sternberg, 1999; Gong et al., 2016), is one of the most important topics in the study of psychology. Creativity plays a vital role in individual career success and represents an unconventional way of thinking that is critical to problem solving, individual progress, change, and innovation (Ward et al., 2008; Tyagi et al., 2017). To fully tap the creative potential of individuals, researchers have explored and validated various factors that may affect creativity, such as individual factors—including personality traits and intelligence (Nusbaum and Silvia, 2011; Simonton, 2014; Barbara et al., 2018; Kenett et al., 2018)—and environmental factors, including family environment and cultural background (Peijia et al., 2006; Lew and Cho, 2013). Thus, most previous studies on creativity have focused on the influence of relatively stable factors.

In recent years, social psychologists have gradually turned their attention to the effect of relatively manipulable cognitive factors on boosting creativity. For example, changing individual cognitive styles can boost creativity; studies have shown that priming one’s own multiple social identities can effectively boost creativity (Gaither et al., 2015). Additionally, usage of stereotypes—an important social cognitive style (Song and Zuo, 2016; Zhang et al., 2016) that implies fixed viewpoints and opinions on the characteristics a certain group (Zuo et al., 2006, 2018)—is also receiving increased attention by social psychologists and is emerging as a new way of boosting creativity. Hudson (1968) first attempted to use stereotypic information to boost the creative potential of elementary school students. The study asked participants in the experimental group to imagine themselves as “eccentric poets,” while participants in the control group were asked to imagine themselves as “hardworking scientists.” The results of this study showed that the experimental group performed better than the control group in a subsequent divergent-thinking task. On this basis, Dumas and Dunbar (2016) used a multi-purpose task to measure the divergent-thinking performance of participants and found that both fluency and originality of the divergent thinking of participants in the group primed with low-creativity stereotypes (e.g., “a stubborn librarian”) were lower than that of participants primed with high-creativity stereotypes (e.g., “an eccentric poet”); that is, the effect of primed stereotypic information on creativity was based on the connectivity between stereotypic content and creativity. Later, de Rooij et al. (2017) attempted to manipulate the relationship between the participants’ network avatars and their corresponding creativity in a 3D virtual environment. They asked the participants to imagine themselves as the avatar and to generate creative examples of objects characterized by a particular feature, which was used to measure participants’ creativity. The results showed that non-creative network avatars (worker stereotypes) reduced creativity, while creative network avatars (artist stereotypes) had no significant effect on creativity. All of the studies above focusing on the effect of stereotypic information on creativity came to a relatively consistent conclusion, which is that priming stereotypes with a strong connection to creativity (e.g., “an eccentric poet,” “artists”) could improve one’s creativity or at least keep creativity constant, while priming stereotypes with little connection to creativity (e.g., “a stubborn librarian,” “office workers”) would reduce creativity. It can be seen that the creative connectivity of priming information—that is, the degree of association between social groups/professional roles and the perceived creativity of these groups/roles—plays an extremely important moderating role in how stereotypes affect creativity.

In addition to exploring the impact of stereotypically consistent information (abbreviated as SCI, which means information that corresponds to existing stereotypes), recently, the effect of the priming with stereotypically inconsistent information (abbreviated as SICI, which means information that does not correspond to existing stereotypes) on creativity has aroused great interest among social psychologists. For example, Gocłowska et al. (2013) conducted two experiments to investigate this effect. In this paper, Study 1 used an “unintentional plagiarism” task and found that priming with SICI could effectively stimulate participants’ cognitive flexibility and reduce their reliance on the availability of knowledge. Study 2 asked participants in the stereotypical condition to come up with five social combinations that “should go together,” while participants in the stereotypically inconsistent condition were asked to think of five social combinations that “should not go together.” Afterward, the researchers used a poster-design experiment to measure creativity, which required the participants to generate new ideas for a themed night at a college club and display their ideas on posters. The results showed that priming with SICI could boost the performance of the participants in the creative task, even if the priming information was unrelated to any specific target group. For this result, Gocłowska et al. (2013) argued that when SICI was primed, stereotyped or schematized knowledge would no longer be effective due to participants’ less frequent reliance on available knowledge. Thus, priming with SICI is beneficial to boost the flexibility and creativity of individual thinking. Based on these results, researchers (Gocłowska et al., 2014) further explored the boundary conditions of the creativity-boosting effect. They found that the boosting effect only existed in individuals with a low personal need for structure (PNS). PNS is defined as “the long-term tendency to create and use abstract mental representations (such as pictures, scripts, attitudes, and stereotypes) that have been simplified by previous experience” (Sun et al., 2016). When exposed to SICI, low PNS promoted participants’ divergent thinking, making them perform better on insight problem-solving, but it had no effect on aggregate thinking. Although high PNS did not affect the individuals’ aggregate thinking, it inhibited their divergent thinking, making them perform worse when solving insight problems. Later, Damer et al. (2018) found that need for cognition (NFC), which refers to the tendency of a person to participate in and enjoy cognitive activities that require effort, also influenced the effect of SICI on cognitive flexibility. For individuals with low NFC, exposure to SICI increased their cognitive flexibility. Conversely, for individuals with high NFC, SICI was not enough to surprise them and, thus, would instead reduce their cognitive flexibility. Therefore, according to these previous findings, it can be inferred that the effect of SICI on creativity is moderated by multiple variables. However, the existing literature has mostly explored the boundary conditions of the boosting effect of SICI on creativity from the perspective of evaluators’ individual differences (such as PNS and NFC), while few studies have examined the possible moderating role of priming information—such as its creativity connectivity—in the creativity-boosting effect of SICI. Therefore, this study will further examine the creativity-boosting effect from this information-priming perspective.

Furthermore, previous studies on the boosting effect of SICI on individual creativity have mostly been carried out in a Western context, whereas Confucianism—which is dominant in China (Liu, 2018)—advocates a moderation-thinking pattern and an educational system that promotes collectivist culture, which is extremely different from the framework of Western countries. Hence, it is necessary to examine whether the positive effects of SICI on creativity also exist in this Eastern kind of social context. In addition, the moderating role of the creative connectivity of priming information in the creativity-boosting effect is also worth exploring further. Therefore, this study first examined whether the positive effects of SICI on creativity still exists in the context of Chinese culture and then, in Study 2, explored the moderating role of creative connectivity of priming information.

## Study 1: The Effect of Stereotypically Inconsistent Information on Creativity

### Purposes and Hypotheses

We aimed to use the priming paradigm and the poster paradigm to explore whether the priming of SCI/SICI would have a boosting effect on creativity. The poster paradigm has previously been used to measure creativity by Gocłowska et al. (2013). In this paradigm, participants are asked to think of a party theme for a college club and to display their ideas on posters. Then several raters—blind to the experimental conditions and hypotheses—judge the creativity of ideas and posters. When the inter-rater reliability is acceptable, the average score of the raters is used as the participant’s creativity score. In this study, we hypothesized that, compared with SCI priming, SICI priming would boost the creative performance of participants more effectively.

### Methods

#### Participants

The required test volume for calculations using G^∗^Power 3.1 was 89 people (assuming a significance-level α of 0.05, a statistical power (1 - β) of 0.95, and an effect size of 0.45). A total of 89 students (41 males and 48 females) with an average age of 19.12 years (*SD* = 1.90) from Wuhan were recruited through advertising in college. All of the participants volunteered to be involved in the study and were compensated with six yuan after completing the experiment. Each subject was randomly assigned to an experimental condition. The results of specific grouping, gender, and age distributions are shown in Table 1.

**Table 1:** Distribution and descriptions of gender and age in each group (*N* = 89).

		SCI priming			SICI priming		
			
Participant gender	Target gender	*n*	*M_age_*	*SD*	*n*	*M_age_*	*SD*
Male	Male	11	20.0	2.49	10	19.9	1.85
	Female	10	20.0	2.36	10	20.7	2.21
Female	Male	13	17.9	0.56	12	18.4	0.79
	Female	10	18.7	1.49	13	18.2	0/99


In addition, this study was carried out in accordance with the recommendations of APA ethical guidelines. The protocol was approved by the Ethics Committee of the Research Center for Social Psychology at Central China Normal University. Before the experiment, all subjects gave written informed consent in accordance with the Declaration of Helsinki. The informed consent included a brief description of the study and potential risks. Subjects were also informed of the experiment duration, their right to withdraw from the experiment at any time, the confidentiality and anonymity protection of their data, and the contact information of the lead researcher. Participants indicated their willingness by checking the “I agree” option and then moved on to the experiment. This informed consent procedure was identical in Study 2.

#### Experimental Design

The experiment adopted a 2 × 2 group design. The independent variables were the information’s target gender (male vs. female) and priming type (SCI priming vs. SICI priming), which were both between-subject variables, while the dependent variable was the performance of the participants in the poster design.

#### Selection of Experimental Materials

First, we chose “a male governor” and “a female nurse” as the targets of the SCI group, and “a female governor” and “a male nurse” as the targets of the SICI group. Then we used a subjective evaluation method to test the validity of the experimental materials. Specifically, we recruited 37 participants (21 males and 16 females) through the QQ group platform; their average age was 20.73 years (*SD* = 2.16). Participants were asked to complete a questionnaire on the Wenjuanxing questionnaire platform^1^, which was used to rate the typicality of the four targets as a male/female. For example, we asked, “To what extent do you think ‘a male nurse’ is typical among men?” The participants rated them on a seven-point Likert scale (from 1 = “very typical” to 7 = “very atypical”). A high score indicated that the participant thought that the target was atypical and was considered an anti-stereotype, while a low score indicated that the subject thought that the target was typical and was considered a stereotype.

The results of repeated measures ANOVA showed that the mean scores of the two targets in the SICI group (*M* = 4.76, 4.35) were significantly higher than those of the two targets in the SCI group (*M* = 2.41, 2.54; *p* < 0.001 for both). Hence, the experimental materials were validated.

#### Experimental Procedure

This experiment was divided into two phases, namely, the priming phase and the creativity-measurement phase. First, participants completed the SCI/SICI priming phase through an adjective description task (Leicht et al., 2014), which required participants to use six adjectives (trying to avoid repetition) to describe a specific target. The target for the SICI priming group to describe was “a male nurse” or “a female governor,” while the target for the SCI priming group to describe was “a male governor” or “a female nurse.” For example, the instruction was, “Please use six adjectives to describe ‘a male nurse’.” After the description task, each participant completed a manipulation check to ensure the validity of the independent variable, that is, the priming type. The corresponding manipulation-check question for each condition was the same as in the pre-experiment.

The second phase took place immediately after the manipulation check, during which we used poster design (Gocłowska et al., 2013) for creativity evaluation. All participants were asked to design a poster for a party at a college in 5 min, and were instructed to make the poster as novel and unique as possible. After collecting all the posters, we invited three raters—who were blind to the purpose of the experiment—to evaluate the novelty and creativity of the posters using a five-point Likert scale (from 1 = “very uncreative” to 5 = “very creative”). Then we calculated the inter-rater reliability of the three scorers to ensure the credibility of the evaluation. A higher score indicated better creativity performance.

### Results

We tested the validity of the independent-variable manipulation using SPSS 21.0. *Post hoc* multiple comparisons of the one-way ANOVA showed that the mean scores of the two targets in the SICI priming group (*M* = 5.55, 3.95) were both significantly greater than those of the two targets in the SCI priming group (*M* = 2.35, 2.08; *p* < 0.001 for both), which indicated that participants in the SICI group thought the targets were more counter-stereotypic than those in SCI group. Hence, our manipulation on priming type was validated.

With regard to the inter-rater reliability of the three raters, we obtained α = 0.76, which indicated that the three scorers’ evaluations on the posters were reliable; therefore, a mean value could be calculated for further data analysis. We conducted a one-way ANOVA on the novelty scores of the participants’ posters. The results showed that the main effect of the priming type was significant [*F*(1,85) = 6.74; *p* < 0.05*; η^2^* partial = 0.06]. The main effect of the target gender was not significant [*F*(1, 85) = 0.08; *p* = 0.78]. Additionally, the interaction between the priming type and target gender was not significant [*F*(1,85) = 2.62; *p* = 0.11]. Specifically, the creativity scores of participants under the condition of SICI priming (*M* = 2.76, *SD* = 0.14) were higher than those of participants under the condition of SCI priming (*M* = 2.25, *SD* = 0.14). This result indicates that, in comparison to SCI priming, SICI priming boosts creative performance significantly better, which confirms our research hypothesis. Meanwhile, the gender of the targets did not have a significant effect on the creativity performance of participants. Specifically, there was no significant difference in the creativity scores of the participants when priming with stereotypically inconsistent men (*M* = 2.94, *SD* = 0.85) vs. when priming with stereotypically inconsistent women (*M* = 2.57, *SD* = 0.96; *p* = 0.18). There was also no significant difference in the creativity scores of the participants when priming with stereotypical males (*M* = 2.13, *SD* = 0.71) vs. priming with stereotypical females (*M* = 2.38, *SD* = 1.11; *p* = 0.38). The specific results are shown in Table 2.

**Table 2:** The results of the one-way ANOVA on the novelty scores of the participants’ posters.

Variation source	*df*	*F*	*p*	η^2^_P_
Priming type	1	6.74^∗^	0.001	0.00
Target gender	1	0.08	0.78	0.07
Priming type × target gender	1	2.62	0.11	0.03


*^∗^p < 0.05.*

### Discussion

Study 1 manipulated the independent variables by asking the participants to write adjectives to describe stereotypically consistent or stereotypically inconsistent individuals, and examined the influence of the priming type on creativity performances. The results confirmed that the priming type can indeed influence the creativity performances of participants; that is, compared to participants who were primed with SCI, participants who were primed with SICI were more likely to think unconventionally and obtained higher scores on the subsequent creativity tests (i.e., poster designs). Further examination about the influence of the gender of the evaluation target on the relationship between the priming type and the creativity score suggested that whether the stereotypically inconsistent target used for priming was male or female did not make a difference, indicating that the positive influence of SICI is not affected by the gender of the evaluation target. In general, the results of Study 1 have confirmed the positive effect of SICI on individual creativity. Next, Study 2 explores the boundary conditions of SICI on creativity-boosting from other perspectives.

## Study 2: The Moderating Role of the Creative Connectivity of SICI

### Purposes and Hypotheses

Study 2 further examined whether priming information with different levels of creative connectivity would have different effects on creativity-boosting. Specifically, we examined whether SICI highly connected with creativity or lowly connected with creativity would have a similar effect on creativity performances. In Study 2, our hypothesis was that, relatively speaking, SICI highly connected with creativity would not boost the creativity of participants while SICI lowly connected with creativity would boost the creativity of participants.

### Methods

#### Participants

We recruited a total of 85 students (41 males and 43 females) from Wuhan through advertising in college. All of the participants were volunteered to be involved in the study and were compensated with six yuan after completing the experiment. Their average age was 19.36 years (*SD* = 1.34), and one of the participants did not write down his or her gender or age. Each subject was randomly assigned to an experimental condition. The specific grouping, gender, and age distribution are shown in Table 3. In addition, this study was also carried out in accordance with the recommendations of APA ethical guidelines. The protocol was approved by the Ethics Committee of the Research Center for Social Psychology at Central China Normal University. All subjects gave written informed consent in accordance with the Declaration of Helsinki. The informed consent procedure was identical for Study 1.

**Table 3:** Distribution and descriptions of gender and age in each group (*N* = 85)^∗^.

		SCI priming			SICI priming		
			
Target gender	Creative connectivity	*n*	*M_age_*	*SD*	*n*	*M_age_*	*SD*
Male	High	9	19.9	0.67	11	19.1	1.04
	Low	10	19.1	1.45	11	19.5	1.13
Female	High	10	20.1	1.60	12	20.2	1.47
	Low	10	18.5	1.35	11	18.6	1.03


*In Study 2, age and gender variables were not our independent variables, so we decided to keep the data of the one subject who did not specify gender and age. His or her experimental condition was low-connectivity SICI priming condition. His or her data would be included in the subsequent analysis, while his or her information is not shown in Table 3.*

#### Experimental Materials

First, we selected “poet” and “nurse” as targets with different levels of creative connectivity and added stereotypically consistent or stereotypically inconsistent characteristic words to form SCI (“an eccentric poet” and “a female nurse”) and SICI (“a dull poet” and “a male nurse”). Then we used subjective evaluation methods to test the validity of the experimental materials. Specifically, we recruited 33 participants (14 males and 19 females) through advertising on campus. Their average age was 21.3 years (*SD* = 2.88). We asked the participants to rate the typicality of the four targets—“an eccentric poet,” “a dull poet,” “a male nurse,” and “a female nurse”—as poets or a nurses. For example, we asked, “How typical is it for there to be ‘a male nurse’ among nurses?” Subsequently, the participants rated the targets on a seven-point Likert scale (from 1 = “very typical” to 7 = “very atypical”). A high score meant that the participant believed that the stimulus was atypical and belonged to the stereotypically inconsistent category and a low score meant that the participant thought that the stimulus was typical and belonged to the stereotypical category.

The results of repeated-measures ANOVA showed that the mean scores of the two targets in the SICI group (*M* = 5.12, 5.06) were significantly higher than those in the SCI group (*M* = 3.39, 2.45; *p* < 0. 001 for both). Hence, the experimental materials were validated.

### Experimental Design

We used a 2 (creativity-domain connectivity: high vs. low) × 2 (priming types: SCI priming vs. SICI priming) inter-group design. Within this design, the priming information of the high-connectivity SCI condition was “an eccentric poet,” and the low-connectivity SCI condition was “a female nurse,” while the high-connectivity SICI condition was “a dull poet,” and the low-connectivity SICI condition was “a male nurse.” The dependent variable was the creativity performance of the participants in the poster design.

### Experimental Procedure

First, we completed the priming phase through an adjective-description task. The target that the participant needed to describe was “an eccentric poet,” “a dull poet,” “a male nurse,” or “a female nurse.” Apart from the descriptive targets in the priming phase, the remaining specific processes were the same as in Study 1. Next, the validity of the manipulation of the independent variables was tested; the specific process was the same as in the pre-experiment. Finally, we measured the dependent variable. We also used the poster-design paradigm and scored the collected posters. The specific process was the same as in Study 1.

### Results

We tested the validity of the independent-variable manipulation using SPSS 21.0. *Post hoc* multiple comparisons of the one-way ANOVA showed that the mean scores of the two targets in the SICI group (*M* = 5.48, 4.61) were significantly higher than those of the SCI group (*M* = 2.65, 3.47; *p* < 0.01 for both). This indicated that the manipulation of the independent variable of priming type was validated.

**FIGURE 1:**
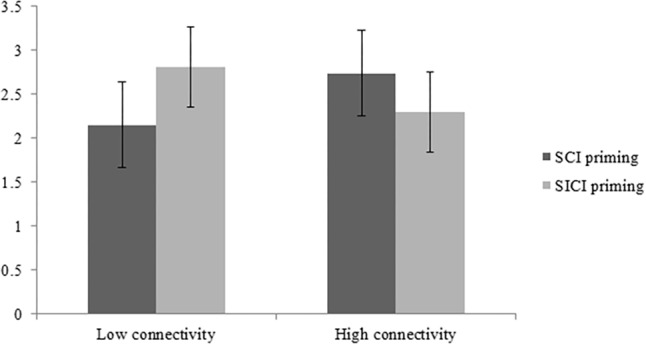
Interaction diagram between the priming type and creativity-domain connectivity.

With regard to inter-rater reliability of the three raters, we obtained α = 0.75, which indicated that the evaluations of the three evaluators were reliable; therefore, a mean value was calculated for further data analysis. We conducted a one-way ANOVA on the novelty scores of the participants’ posters. The results showed that the main effect of the priming type [*F*(1,80) = 0.24; *p* = 0.63] and the main effect of creativity-domain connectivity [*F*(1,80) = 0.03; *p* = 0.87] were not significant, but the effects of the interaction between the priming type and connectivity were significant [*F*(1,80) = 5.23; *p* < 0.05; *η^2^* partial = 0.06]. The specific results are shown in Figure 1.

Since the effects of the interaction between creative connectivity and priming type were significant, we further performed a simple-effect analysis. The results showed that under the high-connectivity conditions, the creativity scores of the participants in the SCI priming group and SICI priming group did not differ significantly [*F*(1,39) = 1.64; *p* = 0.21]. However, under low-connectivity conditions, the creativity scores of the participants in the SCI priming group and SICI priming group differed significantly [*F*(1,41) = 4.23; *p* < 0.05]. Specifically, under low-connectivity conditions, the creativity scores of the SICI priming group (*M* = 2.81, *SD* = 1.14) were significantly higher than those of the SCI priming group (*M* = 2.15, *SD* = 0.95). Furthermore, under the high-connectivity conditions, the creativity scores of the SICI priming group (*M* = 2.3, *SD* = 1.03) did not differ significantly from those of the SCI priming group (*M* = 2.74, *SD* = 1.14). The specific results are shown in Table 4.

**Table 4:** Further simple-effect analysis.

Variation source	*df*	*F*	*p*	*η2 partial*
Priming type (SCI/SICI)				
Low connectivity	1	4.23^∗^	0.04	0.09
High connectivity	1	1.64	0.21	0.04


*^∗^p < 0.05.*

### Discussion

Study 2 explored the moderating mechanism of the relationship between SICI priming and creativity-boosting. Specifically, it examined whether SICI priming affects individual creative performance differently when the creativity connectivity of the priming information is different. The results show that when the priming information has low connectivity to creativity, the priming of SICI is more stimulating to participants’ creative performance than the priming of SCI. However, when the priming information is highly connected to creativity, there is no significant difference between the two. Specifically, when the priming information has low connectivity to creativity, such as the information about the nurse, priming with SICI (“a male nurse”) boosts participants’ creativity more than priming with SCI (“a female nurse”). Furthermore, when the priming information has high connectivity to creativity, such as the information about the poet, priming with SCI (“an eccentric poet”) and priming with SICI (“a dull poet”) do not affect the creative performance of the participants differently. These results suggest that not all SICI can improve the creativity of participants. Only when the connectivity between priming information and creativity is relatively low will SICI be able to better boost creativity.

## General Discussion

From the perspective of social cognition, this study has deepened our understanding of the effect of SICI on creativity-boosting from two different aspects. First, this study has confirmed that priming with SICI is an effective strategy for boosting an individual’s creative performance in the poster-design task, which is consistent with the results of previous studies conducted in a Western cultural context (Gocłowska et al., 2013). Besides, our findings proved that even in a Chinese cultural context, the positive effect of SICI can also be extended beyond the field of stereotypes. Finally, this study proposed and confirmed the moderating effect of creativity connectivity, which further supplements the research of Gocłowska et al. (2014) on the relationship between SICI and creativity. This result indicates that creativity is not a stable characteristic of an individual, but rather a flexible product of the interaction between the situation and the individual.

The results of Study 1 suggest that SICI priming can boost an individual’s creative performance, and that this effect is unrelated to the gender of the target of the priming information. Whether one primes with SICI of a male or female target, both are able to significantly improve the creative performance of participants. This result is consistent with the results obtained by Gocłowska et al. (2013) using a stereotypically inconsistent linking method. On the other hand, it also shows that, in the context of Eastern culture, SICI can also lead subjects to be less likely to apply stereotypical or schematic knowledge, thus making their cognition more flexible and boosting their creativity performances.

In addition, other studies on the effects of SICI on cognition have also provided indirect evidence for the results of this study. For example, many studies have found that the exposure to SICI can effectively reduce people’s stereotypes and prejudices toward specific groups (Lai et al., 2014; Finnegan et al., 2015). Additionally, there have been other researchers who have shown that priming with anti-stereotypes can influence an individual’s self-concept, that is, reducing the stereotyping of one’s implicit self-concept can make one’s explicit self-concept more flexible (Asgari et al., 2010, 2012). All these studies have suggested that priming with SICI can provide an unconventional atmosphere for individuals in a short time, thus affecting their original cognition. In the present study, creativity performance in the poster-design task was enhanced precisely because the previous description task of SICI activated the creative-thinking process of the subjects.

Some of the results in Study 2 provide indirect evidence to corroborate previous studies regarding stereotypes and creativity. According to Dumas and Dunbar (2016), when the priming stimulus has low-creativity connectivity (e.g., “a stubborn librarian”), it is not conducive to individual creativity. Only when the stimulus has high-creativity connectivity (e.g., “an eccentric poet”)—or at least does not have low creativity—will it boost individual creativity. Similarly, we found that when the high-creativity-connected SCI (“an eccentric poet”) was primed, its boosting effect on creativity was marginally significantly higher than that of priming with low-creativity-connected SCI (“a female nurse”), *p* = 0.09, which is consistent with the results of Dumas and Dunbar (2016). What is more, we further explored the impact of SICI with different creativity correlations on creativity. We found that when the SICI were highly related with creativity—such as “a dull poet,” which is anti-stereotyped since information on poets is generally associated with high creativity—it was not conducive to boosting individual creative performance. However, when the SICI had low connectivity to creativity—such as a male nurse, since information about nurses generally has a low connectivity to creativity—it did boost individual creativity.

In general, based on previous research and theories, the present study made some reasonable inferences and further obtained some meaningful conclusions and findings that can extend and supplement previous findings through strict manipulation and control of variables. However, there are still some limitations to our study. First, the participants selected in the two experiments were all college students. We did not consider other social groups, which could have affected the ecological validity of the study. As a special group, college students have stronger cognitive ability and higher intelligence, relative to other social groups. Studies have shown that individual intelligence and creativity have a moderate level of correlation (Nusbaum and Silvia, 2011). Therefore, in future research, it will be necessary to select other social groups—such as teenagers and children—and different occupational groups to test these hypotheses. Second, while Study 2 explored the effect of creative connectivity on creativity-boosting by selecting “an eccentric poet” and “a dull poet,” and “a female nurse” and “a male nurse” as high/low creative connectivity stereotypically consistent/inconsistent targets—future research may also adopt other groups to conduct repetitive tests, such as the following: “an innovative artist” and “a somber artist”; “a male engineer” and “a female engineer.” Third, although the poets and nurses discussed in this study are two common domains of high/low association with creativity, we still consider that the creativity connectivity may be a continuous variable of varying degrees, while nurse is only a domain of medium relevance. Hence, we suggest that future research could further examine the impact of SICI on creativity performance at different degrees of creativity connection (e.g., high vs. medium vs. low). Finally, further exploration of the duration of the effect of SICI on creativity—that is, whether the positive effect is temporary or chronic—is also an important topic for future research.

Building upon the foundation of previous research, the present study found that SICI has a positive boosting effect on creativity in a Chinese cultural context. Additionally, the present study also explored whether the creative connectivity of priming information has influence on this process. The results show that only when the priming information has low connectivity to creativity does priming with SICI promote the creative performance of participants. In sum, this study replicated previous findings in Chinese culture, and revealed that the creativity boosting effect is affected not only by individual differences but also by priming information itself.

## Ethics Statement

The full name and affiliation of the ethics committed of the present study is the Center for Studies of Social Psychology at Central China Normal University.

## Author Contributions

FW and BZ conceived and designed the analysis, and wrote the manuscript. ZX and JG collected the data.

## Conflict of Interest Statement

The authors declare that the research was conducted in the absence of any commercial or financial relationships that could be construed as a potential conflict of interest.

## Footnotes

1 In China, The Wenjuanxing questionnaire platform (https://www.wjx.cn/) is widely used for posting online questionnaire and collecting data. We first posted the questionnaire on the platform and after collecting all the data, we downloaded them from the platform.
